# Evaluating the impact of naloxone dispensation at public health vending machines in Clark County, Nevada

**DOI:** 10.1080/07853890.2022.2121418

**Published:** 2022-09-28

**Authors:** Sean T. Allen, Allison O’Rourke, Jessica. A. Johnson, Chelsi Cheatom, Ying Zhang, Brandon Delise, Kellie Watkins, Kathleen Reich, Rick Reich, Cassius Lockett

**Affiliations:** a Department of Health, Behavior and Society at the Johns Hopkins Bloomberg School of Public Health, Baltimore, MD, USA; b DC Center for AIDS Research, Department of Psychological and Brain Sciences, George Washington University, Washington, DC, USA; c Southern Nevada Health District, Las Vegas, NV, USA; d Trac-B Exchange, Las Vegas, NV, USA

**Keywords:** Public health vending machines, harm reduction vending machines, overdose fatalities, naloxone

## Abstract

**Introduction:**

Implementing public health vending machines (PHVMs) is an evidence-based strategy for mitigating substance use-associated morbidity and mortality *via* the dispensation of essential supplies to people who use drugs, including overdose prevention resources. PHVMs have been implemented throughout the world; however, their implementation in the United States (US) is a recent phenomenon. In 2017, Trac-B Exchange (a syringe services program in Clark County, Nevada) installed three PHVMs. In 2019, naloxone dispensation was launched at PHVMs in Clark County. The purpose of this research is to examine the extent to which naloxone dispensation at PHVMs was associated with changes in opioid-involved overdose fatalities.

**Methods:**

Monthly counts of opioid-involved overdose fatalities among Clark County residents that occurred from January 2015 to December 2020 were used to build an autoregressive integrated moving averages (ARIMA) model to measure the impact of naloxone dispensation at PHVMs. We forecasted the number of expected opioid-involved overdose fatalities had naloxone dispensation at PHVMs not occurred and compared to observed monthly counts. Interrupted time series analyses (ITSA) were used to evaluate the step (i.e. the immediate impact of naloxone dispensation at PHVMs on opioid-involved overdose fatalities) and slope change (i.e. changes in trend and directionality of monthly counts of opioid-involved overdose fatalities following naloxone dispensation at PHVMs).

**Results:**

During the 12-months immediately following naloxone dispensation at PHVMs, our model forecasted 270 opioid-involved overdose fatalities, but death certificate data indicated only 229 occurred, suggesting an aversion of 41 deaths. ITSA identified a significant negative step change in opioid-involved overdose fatalities at the time naloxone dispensation at PHVMs was launched (B = −8.52, *p* = .0022) and a significant increasing slope change (*B* = 1.01, *p*<.0001). Forecasts that extended into the COVID-19 pandemic suggested worsening trends in overdose fatalities.

**Conclusion:**

Naloxone dispensation at PHVMs was associated with immediate reductions in opioid-involved overdose fatalities.

**Key Messages**
- Naloxone dispensation at PHVMs was associated with immediate reductions in opioid-involved overdose fatalities.
- Communities should consider implementing public health vending machines in efforts to prevent opioid-involved overdose fatalities.
- The COVID-19 pandemic worsened the overdose crisis.

## Introduction

Overdose fatalities have increased in the United States (US) for several years [1]. More than 100,000 overdose fatalities are estimated to have occurred in the US during the 1-year period ending in April 2021, representing a nearly 30% increase from the same period in the prior year [2]. Data from the Centres for Disease Control and Prevention (CDC) suggested that opioids were involved in most of these overdose fatalities [2]. Risks for experiencing an opioid-involved overdose are not distributed equally [3–5]. People who inject drugs (PWID) are at elevated risk for overdose (as compared to persons who use drugs *via* non-injection routes of administration) given the rapidity of intoxication following injection drug use [3]. In addition, research has shown that people who use drugs may unknowingly be exposed to potent synthetic opioids (e.g. fentanyl) *via* adulterated drug supplies [6].

In response to the ongoing overdose crisis, communities have implemented an array of overdose prevention initiatives, including: increasing access to evidence-based drug treatment, implementing quick response teams that aim to link persons who recently overdosed to substance use treatment and harm reduction services, passing Good Samaritan laws that encourage persons to seek help during overdose emergencies, and offering fentanyl test strips for drug checking. Programs that distribute the overdose reversal medication naloxone to persons at risk of experiencing or witnessing an overdose have also been implemented and studied among several vulnerable populations [7–15]. Further, a 2016 systematic review found that take-home naloxone program utilisation was associated with reductions in overdose mortality rates [16]. There is also no evidence of naloxone access encouraging or enabling substance use [14, 17–20]. While existing efforts to prevent overdose should not be discounted, sustained escalations in overdose fatalities underscore the need for innovations in how communities approach overdose prevention.

Implementing public health vending machines (PHVMs), sometimes referred to as harm reduction vending machines or syringe vending machines, is an evidence-based strategy for mitigating substance use-associated morbidity and mortality *via* the dispensation of a variety of essential supplies to people who use drugs, such as: sterile syringes and other injection equipment, HIV test kits, condoms, sharps containers, information about available health and social services, first aid kits, hygiene kits, pregnancy tests, and overdose prevention resources [21–25]. PHVMs are not new to the field of public health; they were first implemented in 1987 in Copenhagen, Denmark, and have subsequently been implemented in more than 14 countries [25]. Research has shown that the implementation of PHVMs increases access to sterile injection equipment and reduces syringe sharing behaviours [22]. PHVMs may be particularly effective for reaching populations who may not access services at syringe services programs (e.g. younger PWID) [21].

Although PHVMs have been implemented in many countries, their implementation in the US is a relatively recent phenomena [26–28]. In 2017, Trac-B Exchange (a syringe services program in Clark County, Nevada) installed three PHVMs in efforts to prevent outbreaks of HIV and viral hepatitis among PWID [28]. Initially, the PHVMs at Trac-B Exchange offered persons sterile injection equipment. In 2018, with assistance from Trac-B Exchange, a non-profit organisation (Impact Exchange) was legally established and assumed responsibility for harm reduction services and operating the PHVMs. Due to familiarity of the name and branding, Trac-B Exchange became a doing business as (DBA) for Impact Exchange. After Impact Exchange assumed the PHVM project from Trac-B Exchange, additional PHVMs were implemented and, in March 2019, naloxone dispensation was made available at three PHVMs in Clark County. Existing public health literature about PHVMs is informative, but no work has examined the extent to which naloxone dispensation at PHVMs in the United States is associated with shifts in overdose mortality. This reflects a significant gap in the literature given that many communities throughout the United States recently implemented PHVMs. Evaluating the degree to which naloxone dispensation at PHVMs in Clark County affected overdose fatalities may provide important insights about the public health utility of PHVM implementation during a national overdose crisis.

## Methods

### Study context

Clark County is located in southern Nevada (US) and spans nearly 8,000 square miles [29]. Though the majority of land space in Clark County is rural, it has a population over 2.2 million that primarily resides in and around Las Vegas [29,30]. Paralleling broader trends in overdose fatalities at the state level, Clark County has been deeply affected by the opioid overdose crisis [31]. For example, in 2020, there were nearly 200 fentanyl-involved overdose fatalities [31]. Overdose prevention initiatives in Clark County have existed for several years, including naloxone dispensation at pharmacies, public health agencies, community-based organisations, and the syringe services program at Trac-B Exchange. In addition, the Southern Nevada Health District (SNHD) provides overdose prevention education and naloxone to first responders and persons who may witness or experience an overdose in Clark County.

### PHVM implementation & operations

Trac-B Exchange first implemented three PHVMs in 2017. Over time, additional machines were implemented in 2018, 2019, and 2020 (three, four, and seven, respectively) throughout Clark County; however, not all PHVMs were operational during a given calendar year. PHVMs were implemented at organisations that served PWID (e.g. offered substance use treatment, clinical services) and expressed a willingness to host a PHVM. In terms of where the PHVMs are geographically located, most operate in the area between downtown Las Vegas and Southeast Las Vegas. All PHVMs in Clark County are located indoors and accessible within the hours of operations of the sites where they operate. PHVMs being located indoors stems from several factors, including: the need to prevent the contents from exposure to extreme temperatures and weather conditions, internet access (to track vends), and reducing wear and tear on the machine. Individuals interested in accessing resources at PHVMs first had to register with Trac-B Exchange. The registration process could be completed at the Trac-B Exchange storefront location or *via* engaging with outreach staff in the community. Once registered, persons received a card with their vending identification number and a magnetic swipe card with their unique identifier pre-programmed on it. Unique identifiers were used by staff to track products dispensed at each PHVM and assist with inventory management. In March 2019, naloxone dispensation was made available at three PHVMs. Each week, naloxone access was limited to one kit (containing two doses) per client. In the 12-months following the launch of naloxone dispensation at PHVMs (and leading up to the global COVID-19 pandemic), more than 1,800 naloxone doses were distributed.

### PHVM operations during the COVID-19 pandemic

At the onset of the COVID-19 pandemic, access to PHVMs was affected differentially based on site. For example, during the early months of the pandemic, one site had staff conduct vends on behalf of clients, potentially creating a higher threshold for service access. Another clinic (which also had a PHVM) required that all persons ring a doorbell to obtain entrance in efforts to mitigate COVID-19 risks. PHVM was less affected at other sites; for instance, one site scaled back service access during the first four months of the pandemic, but later reopened in June 2020. In another venue, access to PHVM was not affected at all by COVID-19 mitigation strategies. The COVID-19 pandemic also created challenges in terms of keeping machines fully stocked. For example, Trac-B Exchange limited its operational hours and, by extension, had less time to restock PHVMs. Additionally, some clients who utilised the PHVMs presented multiple identification cards, potentially reflecting secondary exchange activities (i.e. accessing services on behalf of others) in efforts to reduce COVID-19 risks.

### Overdose fatality data

**Figure 1.**
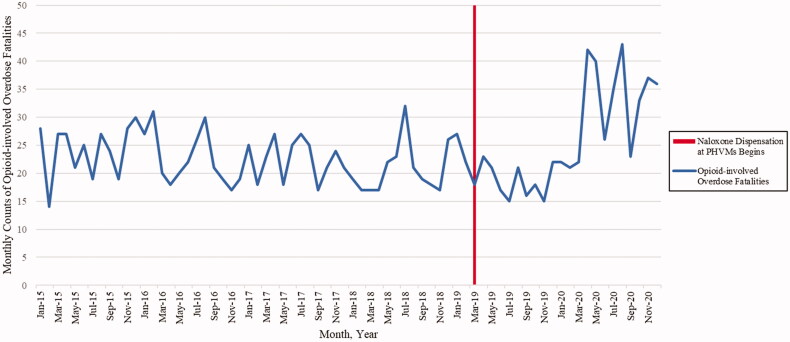
Opioid-involved Overdose Fatalities in Clark County, Nevada, by month (January 2015 to December 2020).

Monthly counts of opioid-involved overdose fatalities that occurred in Clark County and among Clark County residents were obtained using death certificate data from the SNHD. Similar to other overdose fatality analyses, overdose fatalities were identified using the following International Classification of Disease, Tenth Revision (ICD-10) underlying cause of death codes (X40–X44, X60–64, X85, and Y10–Y14) [32,33]. Opioid-involved overdose fatalities were then identified among the overdose fatalities *via* multiple cause-of-death codes that indicated opioids (T40.0, T40.1, T40.2, T40.3, T40.4, or T40.6). Data were organised into monthly counts of opioid-involved overdose fatalities as a time series spanning January 2015 to December 2020. March 2019 served as the interruption in our time series given that it was when naloxone dispensation at PHVMs started (Figure 1). January 2015 to February 2019 reflected the pre-implementation period and March 2019 through December 2020 constituted the post-implementation period.

### Model fitting

We used Autoregressive Integrated Moving Averages (ARIMA) modelling to evaluate the impact of naloxone dispensation at PHVMs [34]. ARIMA models consist of three components: the autoregressive component (the autocorrelation among time points), the moving averages component (refers to each data point as a function of the average of the error terms in a specified number of prior data points), and the integrated component (the trend in the time series) [35,36]. We used ARIMA modelling for this analysis given that it can account for autocorrelation and seasonality in time series data [37]. Further, as compared to segmented regression analyses, conducting interrupted time series analyses with ARIMA models can afford nuanced examinations of complex patterns that result from an intervention [37].

Our ARIMA model was fit to the time series using the Box and Jenkins method [38]. For model identification, autocorrelation plots, the Augmented Dickey-Fuller (ADF) test, minimum information criterion method (MINIC), smallest canonical correlation method (SCAN), and the Ljung-Box test for white noise were used for the entire time period as well as for the pre-intervention period (i.e. pre-PHVM implementation). Model fit was assessed for each of these periods given our interest in understanding the impact of naloxone dispensation at PHVMs and forecasting overdose mortalities without their implementation. The ADF test did not identify a unit root, indicating the data were stationary across both time periods and did not require differencing.

Next, we sought to identify the autoregressive and moving average components of the ARIMA model *via* examining the autocorrelation function (ACF) and partial autocorrelation (PACF) plots of the stationary series (Appendix A). There was a positive autocorrelation at lag 1, which suggested autoregressive terms were needed [34]. Additionally, the ACF plot decayed more slowly than the PACF plot, further suggesting that an autoregressive term was needed and that the time series was slightly under-differenced. The lag at which the PACF plot cut off was three, suggesting that a third order autoregressive term was needed. Taken together, our ACF and PACF plots did not suggest a moving-averages component was necessary. We also used the MINIC and SCAN functions to further assess best potential autoregressive and moving-average orders. We fit multiple iterations of the model to each data set and compared Akaike’s information criterion (AIC), Schwarz’s Bayesian criterion (SBC), Ljung-Box test statistics for white noise, and assessed for outliers before selecting our final model. In essence, we initially considered third order as the most likely identification of the autoregressive component of the ARIMA model, but also compared it to first, second, and fourth order models *via* AIC, SBC, and Ljung-Box test statistics for white noise. These comparisons suggested that a third order autoregressive parameterisation was best fitting to the data. Collectively, our modelling fitting processes indicated an ARIMA (3,0,0) was the best model type to use for our analyses.

### Analyses

The fitted model was used to conduct two types of analyses, interrupted time series analyses (ITSA) and forecasting. This analytic approach (ARIMA modelling and ITSA) has been shown to be a useful strategy for assessing the impact of an intervention on time series data [39–41]. ITSA was used to evaluate step and slope changes in monthly counts of opioid-involved overdose fatalities following naloxone dispensation at PHVMs. The step change (which measured the immediate impact of naloxone dispensation at PHVMs on opioid-involved overdose fatalities) was evaluated *via* a dichotomous variable (0/1) that indicated if a given monthly count of overdose fatalities occurred before or after naloxone dispensation at PHVMs. The slope change (which measured changes in trend and directionality of monthly counts of opioid-involved overdose fatalities following naloxone dispensation at PHVMs) was evaluated using a continuous variable in which all months preceding naloxone dispensation were assigned a value of zero and the month in which naloxone dispensation launched was assigned a value of 1, with subsequent observation values increasing by 1 (1, 2, …, n) until the end of the time series in December 2020.

Using the ARIMA model, forecast analyses were conducted to obtain monthly counts of opioid-involved overdose fatalities that would have been expected had naloxone dispensation at PHVMs not occurred. Given the magnitude of the global COVID-19 pandemic on all facets of society, we compared the forecasted number of overdose fatalities relative to the actual counts over three time periods:

1. the 12-months immediately following naloxone dispensation at PHVMs and preceding the identification of the COVID-19 pandemic (March 2019 to February 2020)
2. the 22-months following naloxone dispensation at PHVMs, inclusive of COVID-19 pandemic-era data (March 2019 to December 2020)
3. the 10-months during the COVID-19 pandemic (March 2020 to December 2020).

For each of the time periods, the observed and expected opioid-related overdose fatalities were compared to calculate the difference between them. All data analyses were conducted using SAS 9.4. Given that this analysis did not constitute human subjects research, it was not reviewed by an Institutional Review Board.

## Results

Between January 2015 and December 2020, Clark County averaged 23.5 opioid-involved overdose fatalities per month, with fewer on average in the months preceding naloxone dispensation at PHVMs (January 2015 to February 2019) than in the post-naloxone dispensation period (March 2019 to December 2020) [22.5 vs. 25.7, respectively; *p*=.0501]. An ARIMA (3,0,0) model was determined to be the best fit for the time series with no outliers or seasonal trend detected during the months preceding naloxone dispensation at PHVMs. During the 12-months immediately following naloxone dispensation at PHVMs (March 2019 to February 2020), the model forecasted 270 opioid-involved overdose fatalities, but death certificate data indicated only 229 actually occurred during this time, suggesting an aversion of 41 deaths. Forecasting for the 22-month period following naloxone dispensation at PHVMs (March 2019 to December 2020) and partially overlapping with the COVID-19 pandemic, the model estimated that 495 opioid-involved overdose fatalities would have occurred; however, death certificate data indicated that 566 actually occurred, a difference of 71 more deaths than expected. Forecasting for the 10-month period that overlapped with the COVID-19 pandemic (March 2020 to December 2020) estimated that 225 opioid-involved overdose fatalities would have occurred, but in actuality, 337 were observed, reflecting an additional 112 fatalities than expected.

Using the same ARIMA (3,0,0) model, interrupted time series analyses were conducted to examine the immediate and trend change in opioid-involved overdose fatalities following naloxone distribution at PHVMs. Results of these analyses can be found in Table 1. The ITSA model detected a significant negative step change in opioid-involved overdose fatalities at the time naloxone dispensation at PHVMs launched (B=-8.52, *p*=.0022) and a significant increasing slope change (*B* = 1.01, *p*<.0001).

**Table 1.** Impact of naloxone dispensation at PHVMs on opioid-involved overdose fatalities in Clark County, NV.

	Coefficient	t-Value	*p* Value
Constant	22.58	26.68	<.0001
Immediate effect of naloxone dispensation at PHVMs	−8.52	−3.18	.0022
Change in trend following naloxone dispensation at PHVMs	1.01	5.26	<.0001

## Discussion

Our analyses suggest that naloxone dispensation at PHVMs in Clark County, Nevada was associated with immediate reductions in opioid-involved overdose fatalities. However, these reductions were situated within a broader context of worsening trends in overdose fatalities. The worsening trends in monthly overdose fatality counts parallel trends throughout the United States and may be explained by a drug supply that is widely contaminated with potent synthetic opioids and adverse health effects associated with the global COVID-19 pandemic (e.g. isolation, diminished access to essential health and human services) [31,42,43]. Further, a 2021 multistate study found that access to drug treatment was significantly disrupted during the COVID-19 pandemic, potentially exacerbating overdose risks among people who use drugs [43].

Our forecast models demonstrated that in the 12-months following naloxone dispensation at PHVMs (and preceding the COVID-19 pandemic), 41 fewer overdose fatalities occurred than what would have been expected. In contrast, our analyses that examine the 22-month period following the launch of naloxone dispensation at PHVMs (and reflecting 10 months of the COVID-19 pandemic) suggested overdose fatalities exceeded that which was forecasted, highlighting the far-reaching effects of the COVID-19 pandemic. It is important to be cognisant that there is no evidence to suggest that providing naloxone at PHVMs resulted in escalations in monthly overdose fatalities; indeed, there is widespread scientific consensus that naloxone provision does not encourage or enable substance use [14,17–20]. Further, naloxone has no addictive potential [44].

Immediate reductions in overdose fatalities following naloxone dispensation at PHVMs nested in the broader context of worsening trends in overdose fatalities underscores the need for communities to utilise all available evidence-based strategies for overdose prevention. Mathematical modelling studies have found that combination approaches for overdose prevention are most effective [45,46]. For example, Irvine *et al.* (2019) found that the implementation of three overdose prevention interventions (take-home naloxone, supervised consumption sites, and opioid agonist therapy) in British Columbia averted more than 60% of the overdose fatalities that were estimated to have happened without their implementation [45]. Future work should be conducted to better understand how to scale up overdose prevention interventions, including *via* expanding access to naloxone at PHVMs, throughout the United States.

To our knowledge, this is the first study that examined the impact of naloxone dispensation at PHVMs in the United States. However, PHVMs are not novel interventions as they were first implemented in Denmark in the late 1980s and since expanded to more than 14 countries [21–23,25,47]. Communities should work expeditiously to implement PHVMs given sustained escalations in the overdose crisis. Further, implementing PHVMs may be a viable strategy for meeting the needs for sterile injection equipment among PWID, and by extension, reduce risks for bloodborne infectious disease acquisition. While there is no silver bullet to preventing substance use-related morbidities and mortalities, PHVMs are another evidence-based tool that can be leveraged to support the public health of people who use drugs.

Throughout PHVM implementation in Clark County, there have been many lessons learned. For example, PHVM placement is a key consideration of implementation. Machines should be located at venues that are easily accessible for clients. Machines also require internet access (to support real-time tracking of supply dispensation) and an electrical outlet, potentially limiting the locations where they may be placed. Additionally, organisations may consider partnering with other agencies that provide services (e.g. drug treatment) to people who use drugs to co-locate PHVMs and ensure access is as low threshold as possible. Agencies interested in implementing PHVMs should also carefully consider associated costs, such as: the costs of purchasing PHVMs, day-to-day operational costs (e.g. internet, electric, software), supplies and packaging materials, and insurance. Conducting needs assessments with potential clients prior to PHVM launch may be beneficial for not only informing the type and volume of supplies needed at each machine, but also where PHVMs should be located. Additionally, communities should consider discussing with local populations of people who use drugs what time(s) PHVMs should be accessible. Tailoring when PHVMs are accessible to the times people who use drugs prefer has the potential to enhance their access and utilisation. Before PHVM implementation, stakeholders should also explore community readiness and assess if trainings about harm reduction services are needed.

Our study has several limitations. First, our interrupted time series analysis only includes one interruption (the launch of naloxone dispensation at PHVMs); however, we know other events, such as the COVID-19 pandemic, occurred during the time period analysed. We did not include an interruption in our analyses for the onset of the COVID-19 pandemic given that the impact of the pandemic evolved over time. Our analyses also do not account for the implementation of pandemic-related mitigation strategies (e.g. social distancing) and associated service access challenges. It is possible that the extent to which these events (the COVID-19 pandemic and associated mitigation strategies) affected the utilisation of PHVMs varied over the study period and by site. It is also plausible that each PHVM serves a different segment of the population of people who use drugs with potentially different levels of willingness to seek supplies at PHVMs during a pandemic. Future studies should characterise the networks of people who use drugs that access supplies at PHVMs and explore their preferences for where and when machines are located. In light of these limitations, we elected to examine our forecast models for monthly counts of opioid-involved overdose fatalities in three ways: the 12-months following the launch of naloxone dispensation at PHVMs (preceding the COVID-19 pandemic), the 22-months following naloxone dispensation at PHVMs (including 10 months that overlapped with COVID-19), and the 10-months during COVID-19. While we cannot determine causality with the available data, the forecasts suggest that naloxone dispensation at PHVMs was associated with significant and immediate reductions in overdose fatalities and that the COVID-19 pandemic was associated with increased overdose mortality.

Another limitation is that our analyses are unable to account for where persons who administered naloxone acquired it. This is an important limitation given that naloxone is available at venues throughout Clark County. Notably, an overdose prevention initiative was launched in 2018 which included efforts to distribute naloxone to community stakeholders at risk for experiencing an overdose or who may be a bystander. Another limitation is that we did not examine shifts in the number of EMS calls for overdose nor overdose-associated hospital visits. It is also plausible that shifts in the drug supply may have differentially affected overdose mortality over the study period. An additional limitation is that we only examined monthly counts of opioid-involved overdose fatalities from 2015 to 2020 due to limitations in data availability. More nuanced analyses may be possible as more recent overdose data become available. Another limitation is that our analyses cannot speak to the totality of interconnected factors that affect overdose fatalities at the community level. Future studies should pair quantitative analyses with complementary lines of qualitative inquiry among people who use drugs to understand the overdose prevention landscape more holistically. Finally, our analyses are limited in that we cannot ascertain the potential impact of simultaneously occurring overdose prevention initiatives; our findings cannot speak to causality. It is conceivable that the reductions in overdose fatalities we identified stem from PHVM implementation in combination with other overdose prevention interventions. Looking ahead, studies should be conducted to determine the constellation of interventions that maximise reductions in overdose morbidity and mortality.

In conclusion, naloxone dispensation at PHVMs in Clark County, NV was associated with immediate reductions in opioid-involved overdose fatalities. In the 12-months following naloxone dispensation at PHVMs (and preceding the COVID-19 pandemic), our model forecasted 270 opioid-involved overdose fatalities, but death certificate data indicated only 229 actually occurred, suggesting an aversion of 41 deaths. Future work should be conducted to better understand how to bring PHVMs to scale throughout the United States given that there are sustained increases in opioid-involved overdose fatalities. Communities should work expeditiously to implement evidence-based overdose prevention interventions, including naloxone dispensation at PHVMs.

## Data Availability

Opioid-involved overdose data that supported the findings of this study are available from the Southern Nevada Health District upon reasonable request.

## References

[1] Mattson CL, Tanz LJ, Quinn K, et al. Trends and geographic patterns in drug and synthetic opioid overdose Deaths – United States, 2013-2019. MMWR Morb Mortal Wkly Rep. 2021;70(6):202–207. 33571180 10.15585/mmwr.mm7006a4 PMC7877587

[2] CDC. Drug Overdose Deaths in the U.S. Top 100,000 Annually. 2021.

[3] Degenhardt L, Bucello C, Mathers B, et al. Mortality among regular or dependent users of heroin and other opioids: a systematic review and Meta-analysis of cohort studies. Addiction. 2011;106(1):32–51. 21054613 10.1111/j.1360-0443.2010.03140.x

[4] Paulozzi LJ, Xi Y. Recent changes in drug poisoning mortality in the United States by urban-rural status and by drug type. Pharmacoepidemiol Drug Saf. 2008;17(10):997–1005. 18512264 10.1002/pds.1626

[5] Rigg KK, Monnat SM. Urban vs. rural differences in prescription opioid misuse among adults in the United States: informing region specific drug policies and interventions. Int J Drug Policy. 2015;26(5):484–491. 25458403 10.1016/j.drugpo.2014.10.001 PMC4397122

[6] Amlani A, McKee G, Khamis N, et al. Why the FUSS (fentanyl urine screen study)? a cross-sectional survey to characterize an emerging threat to people who use drugs in British Columbia, Canada. Harm Reduct J. 2015;12:54. 26577516 10.1186/s12954-015-0088-4 PMC4650899

[7] Bennett T, Holloway K. The impact of take-home naloxone distribution and training on opiate overdose knowledge and response: an evaluation of the THN project in Wales. Drugs Educ Prevent Policy. 2012;19(4):320–328.

[8] Enteen L, Bauer J, McLean R, et al. Overdose prevention and naloxone prescription for opioid users in san francisco. J Urban Health. 2010;87(6):931–941. 20967505 10.1007/s11524-010-9495-8 PMC3005091

[9] Galea S, Worthington N, Piper TM, et al. Provision of naloxone to injection drug users as an overdose prevention strategy: early evidence from a pilot study in New York city. Addict Behav. 2006;31(5):907–912. 16139434 10.1016/j.addbeh.2005.07.020

[10] Gaston RL, Best D, Manning V, et al. Can we prevent drug related deaths by training opioid users to recognise and manage overdoses? Harm Reduct J. 2009;6(1):26. 19781073 10.1186/1477-7517-6-26 PMC2762999

[11] Lewis DA, Park JN, Vail L, et al. Evaluation of the overdose education and naloxone distribution program of the baltimore student harm reduction coalition. Am J Public Health. 2016;106(7):1243–1246. 27077351 10.2105/AJPH.2016.303141 PMC4984772

[12] Piper TM, Stancliff S, Rudenstine S, et al. Evaluation of a naloxone distribution and administration program in New York city. Subst Use Misuse. 2008;43(7):858–870. 18570021 10.1080/10826080701801261

[13] Rowe C, Santos G-M, Vittinghoff E, et al. Predictors of participant engagement and naloxone utilization in a community-based naloxone distribution program. Addiction. 2015;110(8):1301–1310. 25917125 10.1111/add.12961 PMC4503489

[14] Wagner KD, Valente TW, Casanova M, et al. Evaluation of an overdose prevention and response training programme for injection drug users in the skid row area of los angeles, CA. Int J Drug Policy. 2010;21(3):186–193. 19268564 10.1016/j.drugpo.2009.01.003 PMC4291458

[15] Walley AY, Doe-Simkins M, Quinn E, et al. Opioid overdose prevention with intranasal naloxone among people who take methadone. J Subst Abuse Treat. 2013;44(2):241–247. 22980450 10.1016/j.jsat.2012.07.004

[16] McDonald R, Strang J. Are take-home naloxone programmes effective? Systematic review utilizing application of the bradford hill criteria. Addiction. 2016;111(7):1177–1187. 27028542 10.1111/add.13326 PMC5071734

[17] Jones JD, Campbell A, Metz VE, et al. No evidence of compensatory drug use risk behavior among heroin users after receiving take-home naloxone. Addict Behav. 2017;71:104–106. 28325710 10.1016/j.addbeh.2017.03.008 PMC5449215

[18] Tse WC, Djordjevic F, Borja V, et al. Does naloxone provision lead to increased substance use? A systematic review to assess if there is evidence of a 'moral hazard’ associated with naloxone supply. Int J Drug Policy. 2022;100:103513. 34798434 10.1016/j.drugpo.2021.103513

[19] Lintzeris N, Monds LA, Bravo M, et al. Designing, implementing and evaluating the overdose response with take-home naloxone model of care: an evaluation of client outcomes and perspectives. Drug Alcohol Rev. 2020;39(1):55–65. 31774221 10.1111/dar.13015

[20] Seal KH, Thawley R, Gee L, et al. Naloxone distribution and cardiopulmonary resuscitation training for injection drug users to prevent heroin overdose death: a pilot intervention study. J Urban Health. 2005;82(2):303–311. 15872192 10.1093/jurban/jti053 PMC2570543

[21] Obadia Y, Feroni I, Perrin V, et al. Syringe vending machines for injection drug users: an experiment in marseille, France. Am J Public Health. 1999;89(12):1852–1854. 10589315 10.2105/ajph.89.12.1852 PMC1509009

[22] Islam M, Wodak A, Conigrave KM. The effectiveness and safety of syringe vending machines as a component of needle syringe programmes in community settings. Int J Drug Policy. 2008;19(6):436–441. 17766100 10.1016/j.drugpo.2007.07.006

[23] McDonald D. The evaluation of a trial of syringe vending machines in Canberra, Australia. Int J Drug Policy. 2009;20(4):336–339. 18790622 10.1016/j.drugpo.2008.06.004

[24] Cama E, Brener L, Bryant J. Characteristics and attendance patterns of a fixed-site NSP and nearby SVM: the benefits of 24-hour access to sterile injecting equipment. Drugs Educ Prevent Policy. 2014;21(6):476–481.

[25] Islam MM, Conigrave KM. Syringe vending machines as a form of needle syringe programme: Advantages and disadvantages. J Subst Use. 2007;12(3):203–212.

[26] Potera C. An innovative syringe exchange program. Am J Nurs. 2017;117(7):17. 10.1097/01.NAJ.0000520934.96160.36 28644276

[27] Vegas H. Impact exchange vending machines. 2021.

[28] Breitenbach S. Las Vegas offers needle exchange program through vending machines. The Washington post. 2017.

[29] Bureau USC. QuickFacts Clark County, Nevada 2021. https://www.census.gov/quickfacts/clarkcountynevada.

[30] US Census Bureau. Census urban and rural classification and urban area criteria. 2010.

[31] District SNH. Fentanyl deaths increasing in Clark County. 2020. https://www.southernnevadahealthdistrict.org/news-release/fentanyl-deaths-increasing-in-clark-county/

[32] Rudd RA, Seth P, David F, et al. Increases in drug and Opioid-Involved overdose Deaths - United States, 2010-2015. MMWR Morb Mortal Wkly Rep. 2016;65(50-51):1445–1452. 28033313 10.15585/mmwr.mm655051e1

[33] Samhsa’s Center For The Application Of Prevention Technologies. Using International Classification of Diseases (ICD) codes to assess opioid-related overdose deaths. 2018.

[34] Schaffer AL, Dobbins TA, Pearson S-A. Interrupted time series analysis using autoregressive integrated moving average (ARIMA) models: a guide for evaluating large-scale health interventions. BMC Med Res Methodol. 2021;21(1):58. 33752604 10.1186/s12874-021-01235-8 PMC7986567

[35] Biglan A, Ary D, Wagenaar AC. The value of interrupted time-series experiments for community intervention research. Prev Sci. 2000;1(1):31–49. 11507793 10.1023/a:1010024016308 PMC4553062

[36] McCleary R, Hay RA, Meidinger EE, et al. Applied time series analysis for the social sciences. Beverly Hills (CA): Sage Publications; 1980.

[37] Li L, Cuerden MS, Liu B, et al. Three statistical approaches for assessment of intervention effects: a primer for practitioners. Risk Manag Healthc Policy. 2021;14:757–770. 33654443 10.2147/RMHP.S275831 PMC7910529

[38] Box GE, Jenkins GM. Time series analysis forecasting and control. San Francisco, CA: Wisconsin Univ Madison Dept of Statistics; 1970.

[39] Pridemore WA, Snowden AJ. Reduction in suicide mortality following a new national alcohol policy in Slovenia: an interrupted time-series analysis. Am J Public Health. 2009;99(5):915–920. 19299669 10.2105/AJPH.2008.146183 PMC2667837

[40] Ruiz MS, O'Rourke A, Allen ST. Impact evaluation of a policy intervention for HIV prevention in Washington, DC. AIDS Behav. 2016;20(1):22–28. 26336945 10.1007/s10461-015-1143-6 PMC4715855

[41] Ruiz MS, OʼRourke A, Allen ST, et al. Using interrupted time series analysis to measure the impact of legalized syringe exchange on HIV diagnoses in Baltimore and Philadelphia. J Acquir Immune Defic Syndr. 2019;82 Suppl 2(2):S148–s154. 31658203 10.1097/QAI.0000000000002176 PMC6820712

[42] Macmadu A, Batthala S, Correia Gabel AM, et al. Comparison of characteristics of deaths from drug overdose before vs during the COVID-19 pandemic in Rhode Island. JAMA Netw Open. 2021;4(9):e2125538. 34533569 10.1001/jamanetworkopen.2021.25538 PMC8449276

[43] Saloner B, Krawczyk N, Solomon K, et al. Experiences with substance use disorder treatment during the COVID-19 pandemic: Findings from a multistate survey. Int J Drug Policy. 2022;101:103537. 34871945 10.1016/j.drugpo.2021.103537 PMC8602971

[44] ASAM. Public policy statement on the use of naloxone for the prevention of opioid overdose deaths; 2016.

[45] Irvine MA, Kuo M, Buxton JA, et al. Modelling the combined impact of interventions in averting deaths during a synthetic-opioid overdose epidemic. Addiction. 2019;114(9):1602–1613. 31166621 10.1111/add.14664 PMC6684858

[46] Ballreich J, Mansour O, Hu E, et al. Modeling mitigation strategies to reduce Opioid-Related morbidity and mortality in the US. JAMA Netw Open. 2020;3(11):e2023677. 33146732 10.1001/jamanetworkopen.2020.23677 PMC7643029

[47] Islam MM, Conigrave KM. Assessing the role of syringe dispensing machines and mobile van outlets in reaching hard-to-reach and high-risk groups of injecting drug users (IDUs): a review. Harm Reduct J. 2007;4:14. 17958894 10.1186/1477-7517-4-14 PMC2146997

